# Coupling blood flow and neural function in the retina: a model for homeostatic responses to ocular perfusion pressure challenge

**DOI:** 10.1002/phy2.55

**Published:** 2013-08-22

**Authors:** Zheng He, Jeremiah K H Lim, Christine T O Nguyen, Algis J Vingrys, Bang V Bui

**Affiliations:** Department of Optometry & Vision Sciences, University of MelbourneParkville, Victoria, Australia

**Keywords:** Ocular perfusion pressure, oxygen extraction ratio, retinal blood flow, retinal function

## Abstract

Retinal function is known to be more resistant than blood flow to acute reduction of ocular perfusion pressure (OPP). To understand the mechanisms underlying the disconnect between blood flow and neural function, a mathematical model is developed in this study, which proposes that increased oxygen extraction ratio compensates for relative ischemia to sustain retinal function. In addition, the model incorporates a term to account for a pressure-related mechanical stress on neurons when OPP reduction is achieved by intraocular pressure (IOP) elevation. We show that this model, combining ocular blood flow, oxygen extraction ratio, and IOP mechanical stress on neurons, accounts for retinal function over a wide range of OPP manipulations. The robustness of the model is tested against experimental data where ocular blood flow, oxygen tension, and retinal function were simultaneously measured during acute OPP manipulation. The model provides a basis for understanding the retinal hemodynamic responses to short-term OPP challenge.

## Introduction

**Figure 1:**
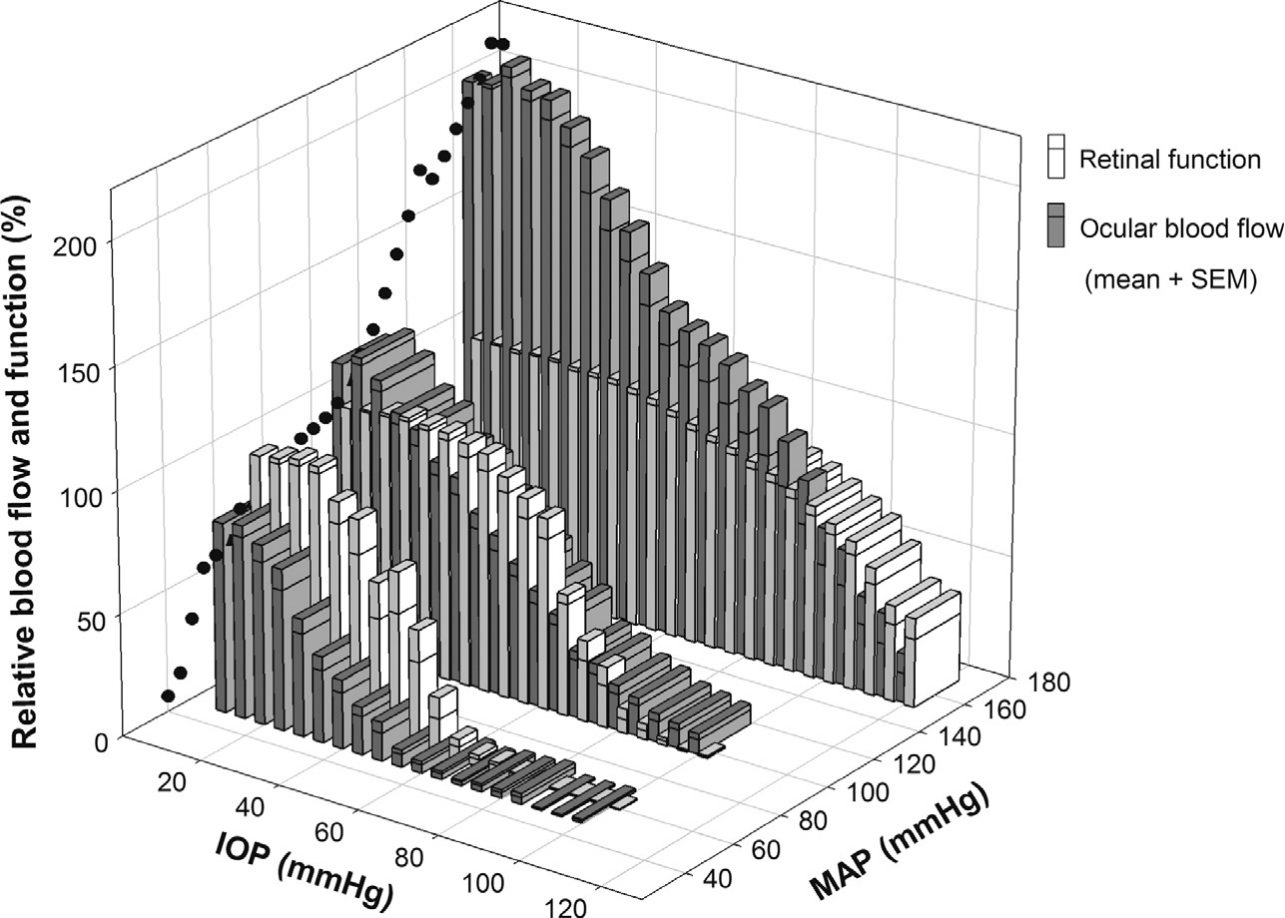
Comparison of retinal function (measured using electroretinography) and blood flow (laser-Doppler flowmetry) during IOP elevation in rats with various blood pressures. IOP was elevated from 10 to 120 mmHg over 60 min (3 min each step of 5 mmHg), during which MAP was held constant at high, moderate or low levels (159 ± 3, 104 ± 3 or 61 ± 1 mmHg respectively). Black circles: blood flow at baseline IOP showing autoregulation in response to changes in MAP. Figure reprinted from a previous study (He et al. 2012; open access, no permission required). IOP: intraocular pressure; MAP: mean arterial pressure; Data are normalized to baseline where 100% represents blood flow and retinal function at IOP of 10 and MAP of 93 mmHg.

Ocular perfusion pressure (OPP) is the balance between mean arterial blood pressure (MAP) and intraocular pressure (IOP), that is*,* OPP = MAP − IOP. Changing OPP, either via MAP or IOP, can alter retinal blood flow, which in turn affects retinal function. Our recent work of short-term perfusion pressure change (He et al. 2012) (Fig. 1) showed that both retinal function and blood flow are progressively attenuated as the level of IOP increases. The importance of blood pressure in this process is highlighted by the increased susceptibility to IOP elevation with low blood pressure, and the converse with high blood pressure. This documented effect was induced by transient blood pressure manipulation (1 h), therefore free of cardiovascular complication associated with chronic hypertension.

During the acute OPP stress shown in Figure 1, there are compensatory mechanisms to maintain retinal blood flow and function to a certain extent. An example of this is illustrated by the black circles in Figure 1 showing that when blood pressure is modified (at the normal IOP of 10 mmHg), blood flow does not simply change in a linear fashion, but shows a relative plateau at MAPs between 60 and 100 mmHg. Outside this range, blood flow changes linearly with MAP. This phenomenon, known as blood flow autoregulation, acts to stabilize blood flow via local regulators of blood vessel diameter (He et al. 2011).

It is of interest that reductions of blood flow (beyond autoregulation) do not always lead to reductions in retinal function, measured as the retina's response to a flash of light (electroretinogram or ERG). This is particularly evident for animals with low blood pressure where the ERG is unchanged even when blood flow has been reduced by more than 50% of baseline (Fig. 1). This suggests that other mechanisms help maintain retinal function even when blood flow autoregulation can no longer cope with OPP reduction. Oxygen extraction ratio (OER) has been proposed to play an important role (Hickam and Frayser 1966; Alm and Bill 1970; Ernest 1974; Linsenmeier and Padnick-Silver 2000). In particular, oxygen extracted per unit arterial blood flow (OER) can be increased to compensate for mild ischemia, thereby maintaining cellular metabolism. An increase in OER may underlie the preservation of retinal function despite reduction in blood flow during OPP challenge (low and normal MAP groups Fig. 1). A reduction in OER would account for our finding that despite hyperperfusion (∼200% blood flow in Fig. 1, acute high MAP) retinal function does not exceed baseline levels. While there is strong evidence in the cerebral circulation that OER changes with blood flow (Fox and Raichle 1986; Hoge et al. 1999), this prospect has yet to be tested in the eye.

**Figure 2:**
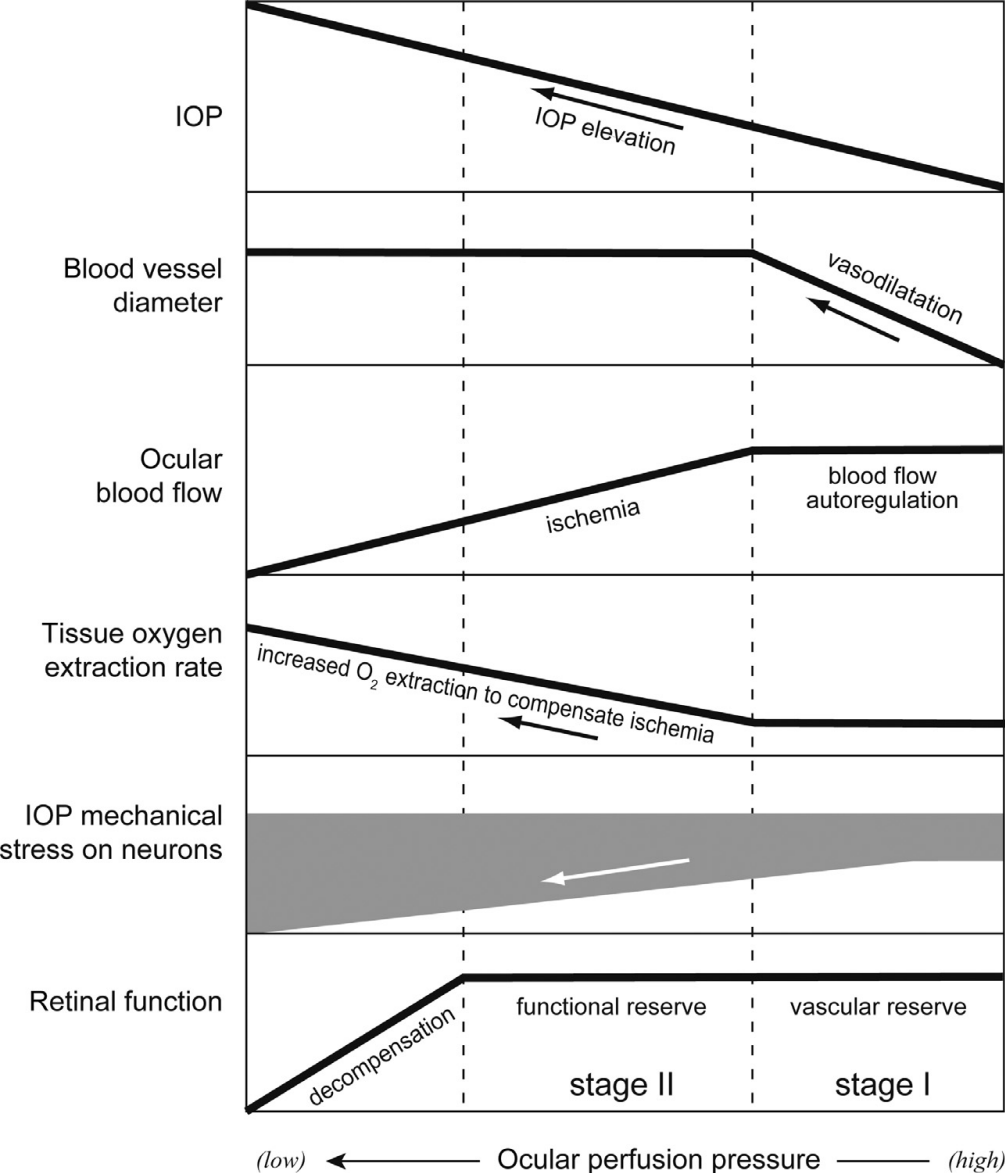
A qualitative description of ocular homeostatic responses to ocular perfusion pressure (OPP) lowering induced by IOP (intraocular pressure) elevation. Compensatory mechanisms that protect against OPP reduction involve two stages. Stage I: vascular reserve is driven by vasodilatation acting to sustain ocular blood flow (blood flow autoregulation). Stage II: retinal functional reserve results from increased oxygen extraction from residual blood flow. If OPP lowering is induced by IOP elevation an additional factor, IOP mechanical stress, also modifies retinal function. Adapted from our review article (He et al. 2011; open access, no permission required).

In addition to the OER as a potential factor to account for the disconnect between retinal blood flow and neural function, other factors may also be important. Compared with the brain, the retina is unique in that changes in IOP have the potential to impact not only on vascular sufficiency (blood flow and OER), but also the neural tissue directly via increased mechanical load (or IOP_M_) on retinal neurons (Maingret et al. 1999; Kalapesi et al. 2005; Kloda et al. 2007) and their axons (Pease et al. 2000). This putative mechanical factor (IOP_M_) could compromise retinal function without affecting blood flow, and thus contribute to the disconnect between blood flow and neuronal function. The contribution of vascular and mechanical stress to IOP-related retinal dysfunction is unclear, as these factors have to date been intractable. We have previously (He et al. 2011) proposed a qualitative model (Fig. 2) that attempts to describe the relationship between retinal ischemia and neural dysfunction as a means to understand the pathophysiology of OPP changes arising from IOP elevation. This qualitative description proposes that oxygen extraction ratio and mechanical stress both increase with IOP elevation. Here, we attempt to quantitatively test these ideas by developing a mathematical model that can characterize the relationship between retinal function, blood flow, oxygen extraction ratio, and mechanical stress. More specifically, we hypothesize that the disconnect between retinal function and blood flow during OPP challenge is driven by changes in oxygen extraction and IOP mechanical stress on neurons. To test this hypothesis, the following aims will be addressed:

1. to develop a model for retinal function based on a subset of the data shown in Figure 1 (the high blood pressure group; we reason that this group provides the widest range of ocular perfusion pressure with which to optimize the parameters of the model).
2. to quantify OER and IOP_M_ from experimental data and compare these against the model predictions.
3. to test the robustness of the model in two separate cohorts of data illustrated in Figure 1 (low and normal blood pressure groups).

## Methods

### Ethical approval

All experimental procedures adhered to the Australian code of practice for the care and use of animals for scientific purposes, published by the National Health & Medical Research Council (NHMRC 2004). Animal ethics approval (Ethics ID: 0705168) was obtained from the Animal Ethics Committee of the Faculty of Science at the University of Melbourne.

To address Aims 1 and 3, a model describing retinal function during OPP challenge was developed based on our recent findings as shown in Figure 1 (He et al. 2012). The methodology for data collection is available elsewhere (He et al. 2012). In brief, MAP in anesthetised Long-Evans rats was held steady for 1 h at either high, moderate, or low levels (159 ± 3, 104 ± 3, and 61 ± 1 mmHg; *n* = 16, 11 and 12, respectively) by intravenous infusion of angiotensin II (1%; 45–90 μg/kg/min), normal saline (5 μL/min), or sodium nitroprusside (2.5%; 50–250 μg/kg/min), respectively. These agents at their respective dosage have been shown to change blood pressure without having a direct drug-related effect on retinal function (see online supplement of He et al. 2012). Simultaneous IOP elevation from 10 to 120 mmHg (in steps of 5 mmHg for every 3 min) was induced manometrically via an anterior chamber cannula. For each blood pressure group, a subset of animals; (*n* = 6 each) was assigned for ocular blood flow assessment (laser-Doppler flowmetry or LDF), whereas the remainder was used to assay retinal function (electroretinography or ERG).

The following section describes the current experimental procedures used to address Aim 2 (quantifying OER and IOP_M_ during OPP reduction).

### General procedures

Animals used in this study were 11 male Long-Evans rats (aged 8 weeks, 300–350 g, Monash Animal Service, Clayton, VIC, Australia), which were housed in a 12-h light (50 lux)/12-h dark environment with normal rat chow and water available ad libitum. All experimental procedures were conducted under general anesthesia (intramuscular injection of ketamine:xylazine mixture, 60:5 mg/kg). Adequate depth of anesthesia was monitored by absence of the paw pinch reflex. A topical anesthetic (proxymetacaine 0.5% eye drops) and mydriatic (0.5% tropicamide) was applied to the eye. Throughout the 1 h experiment, anesthetized rats were placed on a heated platform. Core temperature was measured using a rectal thermometer and remained at 36.1 (95% CI 36.0–36.2) degrees Celsius throughout the experiment. Blood pressure was monitored using a tail cuff sphygmomanometer (ML125, ADIntruments Pty Ltd., Bella Vista, NSW, Australia) and found to remain stable (99 ± 6 mmHg, *n* = 11). While tail cuff sphygmomanometer has limitations, this approach is appropriate when no change in BP is expected and BP is not the primary outcome measure (Kurtz et al. 2005a,b).

### Experimental protocol

Retinal function, blood flow, and tissue oxygen tension (pO_2_) are simultaneously measured over a wide range of acute manometrically controlled IOP levels. As previous (He et al. 2012), IOP elevation from 10 to 100 mmHg (increment of 5 mmHg for every 3 min) was achieved via an anterior chamber cannula connected to a height adjustable reservoir of Hanks balance salt solution (Sigma-Aldrich, Castle Hill, NSW, Australia). The reservoir is connected to a pressure transducer (Transpac®, Hospira, Melbourne, VIC, Australia), which has an accuracy of 0.005 mmHg ± 1%. Analysis of interindividual variation shows that with repeated trails the precision of the set IOP level is 0.587%. Furthermore, a pilot study (online supplement of He et al. 2012) calibrating the stepwise IOP elevation showed that it has a strong linear relationship (slope of 1.0, *r*^2^ = 0.99) with the IOP directly measured from the anterior chamber.

Retinal function was measured with the ERG active electrode (custom-made chlorided silver electrode) placed on the cornea, and a ring-shaped reference electrode positioned around the limbus. The ground needle electrode was inserted subcutaneously in the tail. At each IOP step, a brief stimulus of twenty flashes of luminous energy 2.03 log cd.s.m^2^ at an interstimulus interval of 2 sec was presented on a constant background of 15 cd/m^2^ via a Ganzfeld integrating sphere (Photometric Solutions International, Huntingdale, VIC, Australia). Signals were digitized at 4 kHz with a band-pass filter of 0.3 to 1000 Hz (23 dB). The photopic b-wave was quantified in terms of the trough-to-peak amplitude.

Blood flow and tissue pO_2_ were assayed continuously with a dual LDF and fluorescent fiber-optic oxygen-sensor (BF/OF/E; Oxford Optronix Ltd, London, UK). Using micromanipulators (KITE; World Precision Instruments, Sarasota, FL), the dual-sensor probe was inserted into the vitreous chamber, 1 mm posterior to the limbus. The tip of the probe was advanced to sit just above the retina and away from any major arteries. This location of the probe was confirmed by continuous monitoring of the backscatter parameter.

Ocular blood flow was measured using laser Doppler flowmetry in a manner similar to previous studies (Kiel and van Heuven 1995; Wong et al. 2013). To determine the origin of the LDF signal, a pilot study (He et al. 2012, online supplement) showed that hyperoxemia induced by pure oxygen breathing suppressed the LDF signal by 18%. Therefore, the LDF signal obtained using the current system contains contribution from both retinal and choroidal circulations; hence the terminology “ocular blood flow” is used here.

While intradepth recordings would be ideal to measure tissue pO_2_ across all layers of the retina, in this study we used intravitreal free pO_2_ as a surrogate for proximal retinal oxygenation. This is reasonable because the only source of preretinal vitreal oxygen comes from the inner retina, and the vitreous does not consume oxygen (Wangsa-Wirawan and Linsenmeier 2003). This approach has been shown to be viable in animal models of stress and injury, including hyperoxia challenge (Ernest 1973; Riva et al. 1986), IOP elevation (Ernest 1974), and vessel occlusion (Pournaras et al. 1990). While vitreal pO_2_ can overestimate inner retinal pO_2_, it nevertheless is a useful tool when retinal function is measured simultaneously, as this approach does not require penetrating the retina (Linsenmeier 2012). This is currently the only approach whereby, blood flow, oxygen tension, and retinal function can be measured simultaneously in an in vivo setting with the concurrent control of blood pressure and intraocular pressure. At baseline, the oxygen tension measured in the vitreous was on average 31.5 mmHg (SEM 2.9 mmHg, *n* = 11), consistent with previous reports (32.8–22.6 mmHg) by Alder et al. (1991) and Yu et al. (1999).

### Data analysis

To allow for comparison between blood flow, pO_2_, and retinal function (ERG b-wave), all measurements are expressed as a percentage of baseline condition (IOP = 10 mmHg). This normalization approach allows relative OER and IOP_M_ to be calculated (see Results).

The mathematical models that describe retinal function and its underlying components OER and IOP_M_ were compared with experimental findings. Parameter optimization was achieved by minimizing the sum-of-square merit function between the model and the experimental observation. The 95% confidence interval of the parameters was determined by a bootstrap, which employs Monte Carlo simulation to estimate the distribution of parameters (Effron 1981; Effron and Gong 1983). The goodness-of-fit of the model was qualitatively assessed using a Chi-square (χ^2^) test, which accounts for (1) the discrepancy between observation and model, (2) data variance, and (3) degrees of freedom (df). The Chi-square test returns the probability [*Q* (χ^2^│df)] that the discrepancy arises from chance, with *Q* < 0.001 regarded as a “poor” fit, whereas *Q* between 0.001 and 0.1 is considered “acceptable”; and >0.1 “good” (Press et al. 1992).

## Results

### Developing a model for retinal function during OPP reduction

According to the hypotheses schematized in Figure 2, OPP-induced ERG attenuation is determined by a combination of oxygen consumption and IOP mechanical stress on neurons (IOP_M_), as given by equation 1.


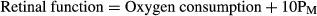
(1)

Normalized baseline retinal function (100% ERG amplitude) is defined as 100% oxygen consumption and 0% IOP-induced mechanical stress (IOP_M_).

### A component describing IOP-induced mechanical stress on neurons

**Figure 3:**
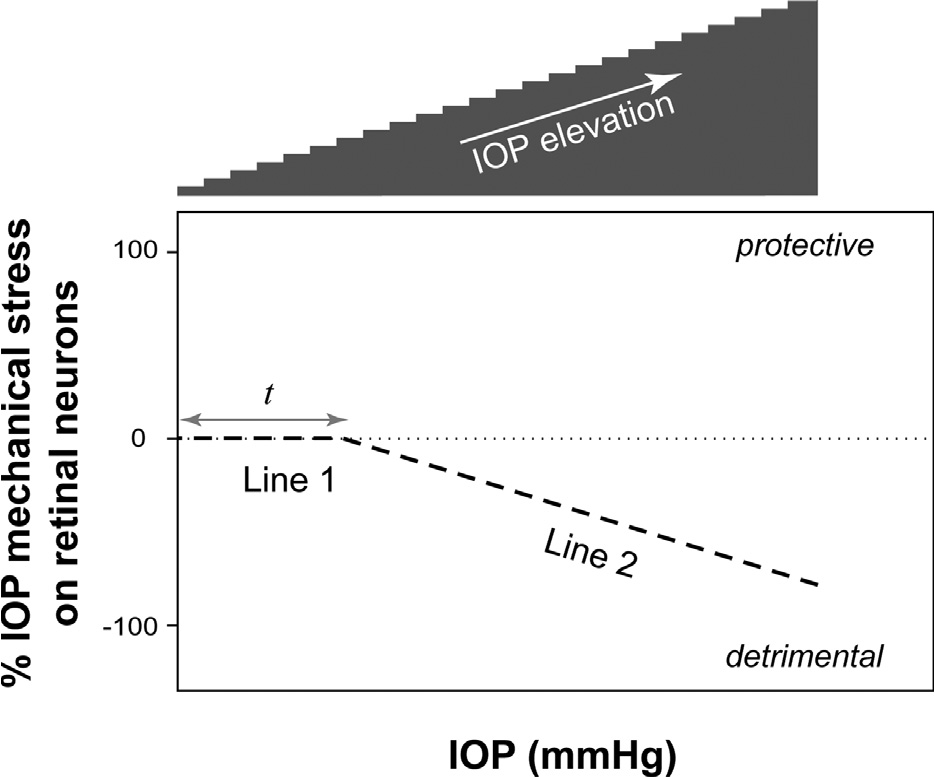
A two-line function (eq. 2) to model the intraocular pressure (IOP) mechanical stress on neurons (IOP_M_) as a function of IOP. The shaded staircase: IOP steps from 10 to 120 mmHg as per experimental protocol in Figure 1. The value of IOP_M_ is ≤0 with progressive IOP elevation, representing a deteriorating effect on retinal function. The width of Line 1 (Parameter “t”) indicates the threshold IOP above which the effect of mechanical stress manifests on neural function.


While it is clear that the mechanical stress and strain experienced by retinal neurons (IOP_M_,%) is dependent on the level of IOP elevation, the form of this relationship (IOP_M_ as a function of IOP) has yet to be defined. We propose that the simplest relationship would be one manifesting a threshold above which IOP_M_ begins to impact negatively upon the ERG (dashed line in Fig. 3; eq. 2).

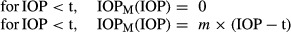
(2)

Equation 2 describes IOP_M_, with a threshold (t) above which ERG compromise becomes apparent. Beyond “t”, IOP mechanical stress modifies the ERG in a linear manner (with a slope of “*m*”). The validity of the two-line function will be further examined by comparing against experimental data as will be described later.

Given that OPP = MAP − IOP, the change in IOP_M_ (eq. 2) can also be expressed as a function of OPP by substituting IOP:


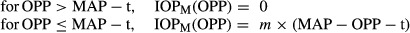
(3)

### A component describing oxygen extraction ratio with changes in blood flow

OER is defined as the ratio between oxygen consumption and delivery (Gibbs et al. 1984; Oja et al. 1999) as given by equation 4. Oxygen delivery can be calculated by the blood flow and the concentration of oxygen in arterial blood [O_2_] (Gibbs et al. 1984).


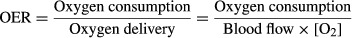
(4)

This function can thus be rewritten as:


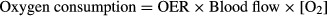
(5)

If we assume that the factors that determine [O_2_] such as hemoglobin concentration and arterial oxygen saturation remain unaltered during acute OPP manipulation (i.e.*,* [O_2_] = 100%), then equation 5 can be simplified as:


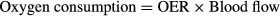
(6)

**Figure 4:**
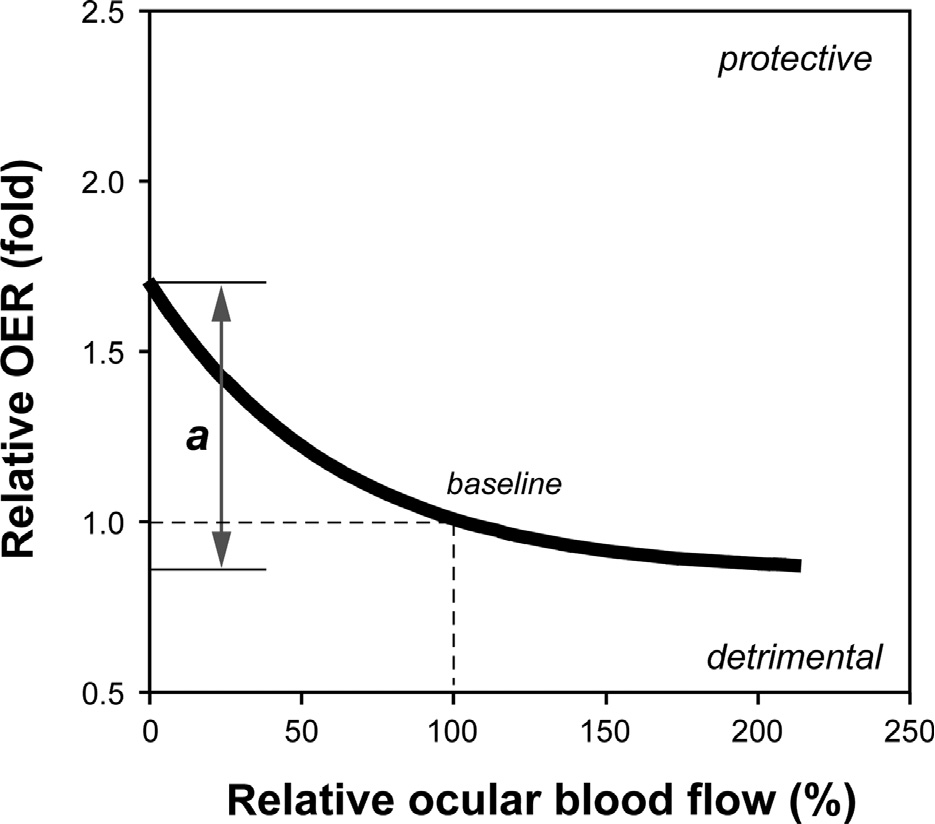
Using equation 7 to model the change of oxygen extraction ratio (OER) as a function of ocular perfusion pressure (OPP). The model is set to pass through baseline (100% blood flow, onefold OER). Double arrow indicates parameter “*a*”, which determines the total magnitude of OER change, that is the OER responsiveness.

Studies from cardiac (Evans et al. 1998) and the cerebral circulations (Buxton and Frank 1997; Hayashi et al. 2003; Guadagno et al. 2006; Andersen et al. 2011) provide strong evidence that, as blood flow slows, OER increases in an exponential manner, as shown in Figure 4 and given by equation 7.


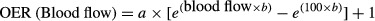
(7)

Equation 7 is a two-parameter exponential function that passes through baseline, which is designated as a relative OER of onefold at 100% blood flow (dashed lines in Fig. 4). An increased OER (>onefold) represents increased oxygen extraction during ischemia thereby offering “protection” for retinal function as it allows for the maintenance of oxidative metabolism. The converse is true for an OER reduction (<onefold). The parameter “*a*” reflects the vertical span of the exponential curve, whereas “*b*” gives the rate of the exponential decay (i.e., half-life = −0.7/b). “*a*” is of physiological interest as it indicates the maximum capacity of a tissue to modify OER in response to changes in blood flow. This might be a useful index of a “functional reserve” to protect against ischemia. A previous study has reported a reduction in “*a*” with cerebral infarction following ischemic stroke (Wise et al. 1983). Equation 7 has been derived based on evidence in the brain. Whether a similar exponential function is also applicable to the eye is not known and will be tested experimentally below. If we assume this to be the case and combine equations 6 and 7, then oxygen consumption can be determined as given by equation 8.


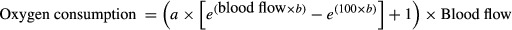
(8)

### Relating blood flow to retinal function during OPP challenge

Equations 8 (oxygen consumption) and 3 (IOP_M_) can be put into equation 1 to provide an overall model relating blood flow to retinal function across a range of OPPs.


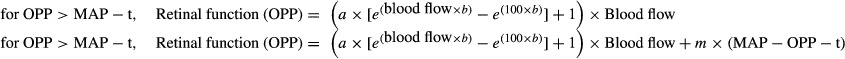
(9)

In equation 9, relative “blood flow” is returned from measured values normalized to baseline measures as per Figure 1. Equation 9 expresses the % change in ERG amplitude as a function of OPP, with parameters “*a*” and “*b*” defining OER, “t” and “*m*” quantifying IOP_M_. Thus equation incorporates the ideas summarized in Figures 2–4 to describe relative retinal function for any given IOP and MAP.

**Figure 5:**
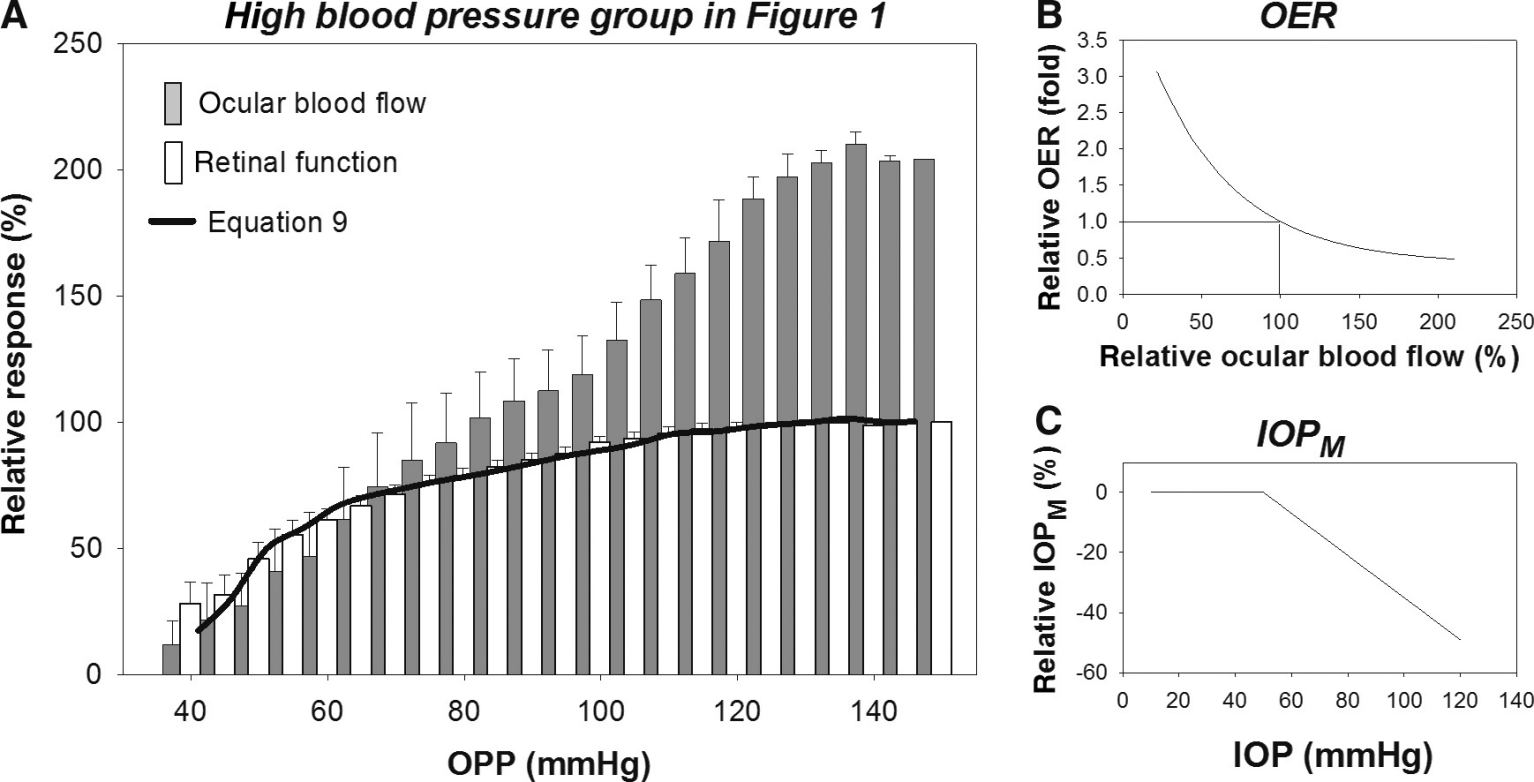
A model (thick trace, eq. 9) relating retinal blood flow and neural function over a range of OPP. (A) Model optimisation is undertaken on experimental data replotted from the high blood pressure group of Figure 1. Parameters “*a*” and “*b*” in equation 9 define the relationship between OER and measured blood flow (B; eq. 7), whereas “t” and “*m*” describe IOP-related mechanical stress (IOP_M_) as a function of IOP (C; eq. 2).

Parameter optimization of equation 9 was achieved by modeling the experimental data recently obtained in acutely hypertensive animals (He et al. 2012), as shown in a subset of Figure 1. This group provides the widest change of ocular perfusion pressure and thus blood flow (200–0%) with which to optimize the parameters of the model. Thus, both relative blood flow and ERG amplitude for this group in Figure 1 are replotted in Figure 5A as a function OPP. By minimizing the sum-of-square merit function between the model (thick trace, eq. 9) and the ERG data (white bars), the optimized parameters given in Table 1 along with the 95% confidence interval are returned. As can be seen in Figure 5A, the model provides an excellent description of changes in % ERG amplitude across a wide range of OPPs. The excellent fit is evident in the Chi-square (χ^2^) statistic, which returns a Q probability of 0.858. In addition to determining the model for retinal function (Fig. 5A), the parameters in Table 1 can also be used to define the contribution of OER (Fig. 5B) and IOP_M_ (Fig. 5C) to retinal function.

**Table 1:** Optimized parameters for the model in Figure 5A (eq. 9)

	*a*	*b*	t	*m*
Best-fit value	4.03	−0.019	50.0	−0.70
95% confidence interval	2.43 to 6.17	−0.024 to −0.013	22.1 to 60.0	−1.16 to −0.32

The above model as expressed in equation 9 makes a number of assumptions. First, we assume that the relationship between OER and blood flow follows an exponential decay (Fig. 5B) in the retina, based on published data in the heart (Evans et al. 1998) and brain (Buxton and Frank 1997; Hayashi et al. 2003; Guadagno et al. 2006; Andersen et al. 2011). Second, we adopt a two-line function to describe the relationship between IOP and IOP_M_ (Fig. 5C), which we reason to be the most parsimonious in the absence of previous data. The following experiment considers the validity of these assumptions by simultaneously measuring blood flow, tissue oxygen tension (pO_2_), and retinal function during IOP elevation, from which we derive the relationship between OER and blood flow, as well as the relationship between IOP_M_ and IOP.

### Measurement of putative OER and IOP_M_

Assuming that the extracted oxygen is fully utilized, the measured tissue pO_2_ can be considered as a surrogate for oxygen consumption. Therefore, according to equation 5, OER can be derived from the ratio of pO_2_ and blood flow:


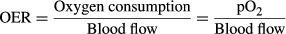
(10)

Making the same assumption, equation 1 can be adapted as:


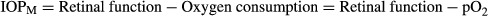
(11)

Thus, equations 10 and 11 allow us to return OER and IOP_M_ from measurements of pO_2_, blood flow, and retinal function. It is important that this validation step employs pO_2_, blood flow, and retinal function data that is simultaneously measured on the same eyes and in a cohort of animals separate to those upon which the model was initially developed (Fig. 5).

**Figure 6:**
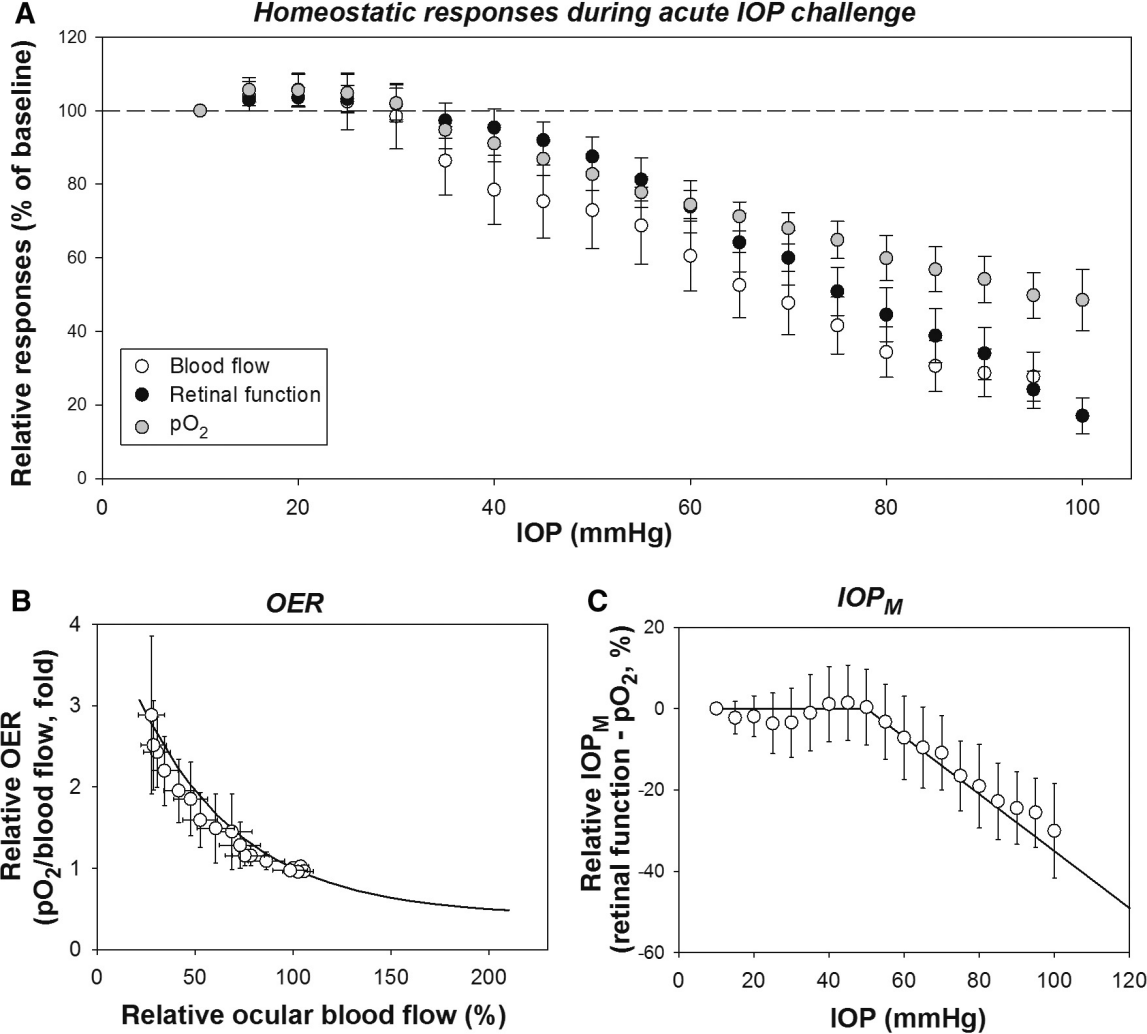
Experimental findings (*n* = 11) to verify the model for oxygen extraction ratio (OER) and intraocular pressure (IOP) mechanical stress on neurons (IOP_M_). (A) simultaneous measurements of relative retinal function, blood flow and pO_2_ (±SEM) during progressive IOP elevation. (B) Calculated from the data in panel A, OER as a function of blood flow (circles) is consistent with previous prediction (curve) reproduced from Figure 5B (parameters “*a*” and “*b*” as per Table 1). (C) The IOP_M_-IOP relationship agrees with the two-line function replotted from Figure 5C (parameters “t” and “*m*” as per Table 1).

Figure 6A shows that relative retinal function (ERG b-wave), blood flow, and pO_2_ change in an IOP-dependent manner. However, the three indices show different susceptibility to IOP elevation (two-way RM ANOVA, interaction term *P* = 0.003).

By expressing all three indices as a relative change from baseline we are able to investigate the relationship between these measures. Specifically, the calculated putative OER derived using equation 9 is plotted as a function of blood flow in Figure 6B. Overlaid with this putative OER measurement is the theoretical OER curve shown in Figure 5B, with parameters “*a*” and “*b*” from Table 1. The observed putative OER-flow relationship is in excellent agreement with the model shown in Figure 5B, as evident by a Chi-square test which returns a *Q* probability of 0.762.

The putative IOP_M_-IOP relationship derived using equation 11 is shown in Figure 6C. The observed IOP_M_ is consistent with the prediction shown in Figure 5C with “t” and “*m*” fixed to the values from Table 1. The theoretical two-line function provides an excellent description of the observed IOP_M_-IOP relationship as evidenced by a *Q* probability of 0.99 (Fig. 6).

### Verifying the model for retinal function

**Figure 7:**
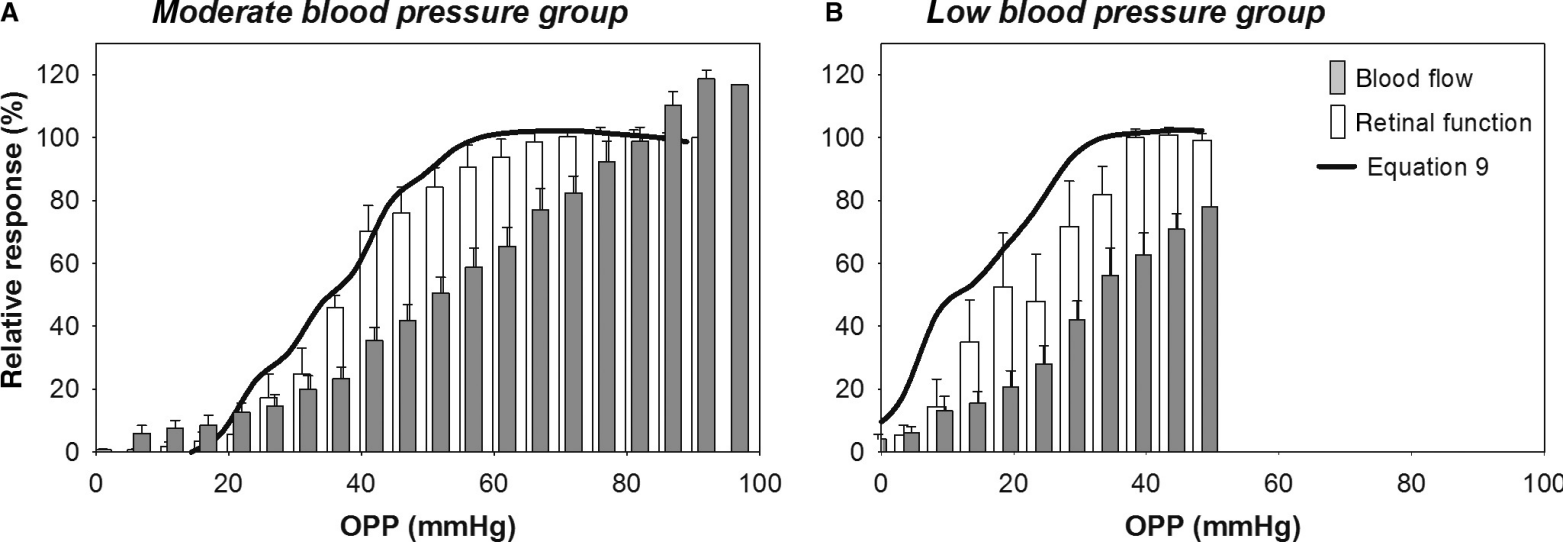
Implementing the model (eq. 9) in rats with moderate (A) and low blood pressure (B). The experimental data are replotted from a previous study (He et al. 2012) as shown in Figure 1. All parameters (“*a*”, “*b*”, “t” and “*m*”) were previously optimised in the high blood pressure group in Figure 1 as given in Table 1.

That the model developed in acutely hypertensive rats (MAP = 159 ± 3 mmHg; Fig. 5B,C) shows a very similar profile to data recorded from a group of normotensive rats (MAP = 99 ± 6 mmHg; Fig. 6B,C) would suggest that the OER-flow and IOP_M_-IOP relationships are independent of blood pressure. Thus it stands to reason that the full model (eq. 9 with parameters given in Table 1) should describe the relationship between blood flow and retinal function for any combination of IOP and MAP. This idea is tested on the moderate (104 ± 3 mmHg) and low blood pressure (61 ± 1 mmHg) groups previously shown in Figure 1. The data for these animals (He et al. 2012) are replotted as a function of OPP in Figure 7A and B, respectively. Inputting the parameters given from Table 1 into equation 9 produces the curves shown in Figure 7. Here, we chose to use these parameters (Table 1; curves in Fig. 6B,C) rather than the experimental data (symbols in Fig. 6B,C) as these parameters were derived from the high blood pressure group, which provides the widest range of OPPs. As a result the full model provides an “acceptable” fit to the ERG data in both moderate (*Q* = 0.03; Fig. 7A) and low blood pressure groups (*Q* = 0.001; Fig. 7B).

## Discussion

This study presents a mathematical model (eq. 9) to describe the relationship between ocular blood flow and retinal function as measured using the ERG in response to changes in OPP. The robustness of the model is supported by the experimental findings that the model developed in one cohort (high blood pressure) can be used to predict responses in other cohorts (moderate and low blood pressures). In addition, the internal consistency of the model was further demonstrated by showing similarities between theoretical and measured putative oxygen extraction ratio (OER, Fig. 6B) and IOP-mechanical stress (IOP_M_, Fig. 6C) components of OPP challenge. To our knowledge, this is the first study to measure retinal function, oxygenation, and ocular blood flow simultaneously during OPP challenge.

### Oxygen extraction ratio in the retina

The use of an exponential decay function to model OER (eq. 7, Fig. 4) was presumed from a similar and well supported model for quantifying the relationship between blood flow and neuronal oxygen metabolism in cerebral circulation (Buxton and Frank 1997; Zheng et al. 2002; Hayashi et al. 2003; Valabregue et al. 2003). The consistency between the OER functions in the retinal and cerebral circulations may be logical as it is well known that the retina and the brain share similar embryological, anatomical, and physiological features (Baker et al. 2008).

To our knowledge, the only previous evidence for increased OER during retinal ischemia was demonstrated by Tornquist and Alm (1979). They showed that the difference between arterial and venous O_2_ saturation, an index of OER, was increased during IOP elevations in pigs, which is qualitatively consistent with the findings in this study in rat eyes. Consistent with this finding, a recent study has found that retinal OER increases in response to hypoxemia induced by low oxygen inspiration in rats (Teng et al. 2013). In humans, a study is currently being undertaken to evaluate retinal blood flow and oxygen extraction in healthy subjects under normal conditions and during hyperoxia (Garhofer 2009). The outcomes of that clinical trial are as yet unpublished.

Our own OER model (Fig. 6B, symbols) is in agreement with data in the cerebral circulation showing that oxygen extraction ratio can be up- or downregulated, which allows buffering against cerebral ischemia (Leenders et al. 1990) or hyperemia (Fox and Raichle 1986; Hoge et al. 1999). This compensatory mechanism appears to be effective only when there is adequate viable cell mass. Wise and colleagues found that the OER responsiveness (parameter “a”) is blunted when cerebral infarction ensues following ischemic stroke (Wise et al. 1983). Indeed, a pattern of reduced oxidative metabolism (neural function) and OER responsiveness has been used as a definition of “established infarction” (Frackowiak 1985), the presence of which influences the choice of treatment strategy in stroke. Therefore, the capacity for OER to change (parameter “*a*”) provides a potentially useful indicator of cellular function and capacity to resist ischemic stress. The possibility that OER curves have the same “a” in disease processes is not known. Nevertheless, the OER model, and in particular its parameter “*a*”, provides a framework for understanding how disease might influence the capacity of the retina to cope with ischemic stress.

### IOP-related mechanical stress

Despite the aforementioned similarity in hemodynamic responses between the brain and the eye, our model (eq. 9) suggests that the retina is unique, in that ocular perfusion pressure gradients are modified by substantial physiological fluctuations in both blood and intraocular pressure. Thus unlike the brain where cerebrospinal fluid pressure is tightly regulated, an important distinction needs to be made when OPP is modified by changing MAP or IOP. It is clear that changes in IOP modify the mechanical load on the retina and more substantially the stress and strain of the connective tissue at the optic nerve head (Burgoyne et al. 2005). As has been previously proposed (Anderson and Hendrickson 1974; Minckler et al. 1977; Johansson 1983), our model presumes that IOP_M_ is solely IOP-dependent; hence a common IOP_M_ function (Fig. 5C) applies to all blood pressure groups. This hypothesis is supported by the findings that the model provides a good fit to various cohorts of rats regardless of their blood pressure levels. This suggests that the vascular and mechanical components of an IOP effect are independent; that is, while blood flow is influenced by the blood pressure of the group, IOP-induced mechanical stress is not. Consistent with this idea, Minckler and co-workers have shown that the IOP-induced axoplasmic stasis in retinal ganglion cells cannot be ameliorated by systemic hyperoxia achieved by pure oxygen breathing (Minckler et al. 1977).

The measurement shown in Figure 6C (along with the model in Fig. 5C) suggests that the critical IOP for mechanical stress to attenuate the ERG in rat eyes is on average 50 mmHg (“t” in Table 1). This threshold is consistent with the data of Johansson (1983) showing that IOP elevation to 50 mmHg causes a significant disruption of ganglion cells axonal transportation. Anderson and Hendrickson (1974) investigated a range of IOPs (10, 15, 55, up to 115 mmHg) in nonhuman primates and showed that the lowest IOP to induce obstruction in axoplasmic flow is 55 mmHg. An in vitro study (Kloda et al. 2007) found that the pressure-sensitive NMDA channels on cultured retinal ganglion cells are activated by pressures above 50 mmHg. Most recently, in vivo imaging of the rat optic nerve head using optic coherence tomography showed that posterior displacement of the optic nerve head structure become evident only when IOP is above 50 mmHg (Zhi et al. 2012). Taken together, these studies suggest that the threshold for significant IOP mechanical stress on neurons is in the order of 50 mmHg.

### Assumptions and limitations

The LDF measurement represents a weighted average of retinal and choroidal blood flow (see online supplement of He et al. 2012), hence the terminology “ocular blood flow” is used here. To compare with the ocular blood flow, the ERG b-wave was chosen as a functional measurement, which is well known to reflect the retinal bipolar cell response (Frishman 2006; Weymouth and Vingrys 2008) and is dependent on both retinal and choroidal blood flow (Fujino and Hamasaki 1965). Therefore, the model in this study cannot differentiate between the retinal and choroidal vasculatures; instead, it should be considered as a model for global vascular and neuronal integrity.

The ERG b-wave (bipolar cell response; Fig. 1) is used over the retinal ganglion cell response as our experiment showed that the greater susceptibility of ganglion cell response is not manifest during the brief step-wise OPP reduction used in this study (He et al. 2012). Instead, the greater susceptibility of ganglion cells to OPP challenge is largely manifest after returning OPP back to baseline (i.e., slower recovery of ganglion cell than bipolar cell response; data not shown).

It is worth noting that the model is developed on data collect by manipulating blood pressure using angiotensin II and sodium nitroprusside (Fig. 1). As these agents are known to modify vascular resistance, local effects in the eye might contribute to the changes in susceptibility to IOP elevation seen in Figure 1. In a pilot study, intravitreal injection of angiotensin II (0.01 mg/mL, 2 μL) in rat eyes, which bypasses any changes in systemic BP, was found not to directly affect ocular blood flow (Z. He, A. J. Vingrys and B. V. Bui, unpubl. data, *n* = 3). In another unpublished pilot study, SNP-induced hypotension (*n* = 6) increased the susceptibility of ocular blood flow to IOP elevation in the same manner as acute exsanguination, a drug free approach that induced the same level of hypotension (*n* = 3). Thus we believe that BP modification rather than local drug effects largely mediates our observed changes in susceptibility of ocular blood flow to IOP elevation.

While the model shows internal consistency across separate groups of rats and is supported by measured data, a number of assumptions were made that are worthy of discussion. First, the model (eq. 8) assumes that O_2_ concentration in arterial blood remains constant. This seems reasonable as one would expect no change in hemoglobin concentration (e.g., anemia) or arterial O_2_ saturation (e.g., respiratory depression) during acute OPP challenge. Second, it is assumed that cerebrospinal fluid (CSF) pressure remains constant during acute OPP challenge. It is known that the CSF pressure will have a significant bearing on the trans-optic nerve head pressure gradient (Morgan et al. 2002, 2008), which can influence mechanical load and thus axonal integrity. Third, it is well known that oxygen and glucose delivery are important for maintaining the metabolic needs of retinal neurons. Our model has not considered the effect of OPP challenge on glucose delivery or uptake. Similar to OER upregulation, an increase of glucose extraction has been reported during retinal ischemia induced by acute IOP elevation (Tornquist and Alm 1979). Therefore, the terminology “oxygen extraction ratio” in this study may actually represent a combined metabolic substrate extraction ratio. Finally, in addition to vascular and mechanical stress, glia cell dysfunction is also suggested as a mechanism leading to IOP-induced injury (Tezel 2009). Thus our terminology “IOP mechanical stress on neurons” may be a misnomer. A better descriptor may be “nonvascular IOP injury” to account for potential mechanical stress on both neurons and glial cells.

## Summary

This study set out to understand the disconnect between neural function and blood flow in the retina during acute OPP challenge, as shown in Figure 1. Using mathematical modeling, we showed that the decoupling between retinal function and ocular blood flow can be accounted for by the capacity of the eye to change oxygen extraction ratio and a nonvascular, pressure-related neuronal dysfunction. The model provides a quantitative description relating blood flow to retinal function for any combination of intraocular pressure and blood pressure. By simultaneously measuring blood flow, oxygen extraction, and retinal function, we show that the eye has a similar OER profile to the brain. Moreover, there is likely to be an IOP threshold above which the effect of IOP-related mechanical stress increases in a linear manner. We believe that our model provides a more comprehensive framework for quantifying the capacity of the eye to buffer ischemia during OPP stress. For the first time, the model allows for the quantification of relative vascular and mechanical stress at any given intraocular pressure and blood pressure. This framework may be particularly useful for studies of glaucoma whereby vascular and mechanical contributions to the death of retinal ganglion cells have thus far been intractable. Moreover, this type of modeling approach may help determine the mechanisms by which systemic risk factors, such as older age, diabetes, chronic hypertension, and myopia contribute to increased risk of ganglion cell loss.
